# Short Interfering RNA Inhibits Rift Valley Fever Virus Replication and Degradation of Protein Kinase R in Human Cells

**DOI:** 10.3389/fmicb.2016.01889

**Published:** 2016-11-24

**Authors:** Bonto Faburay, Juergen A. Richt

**Affiliations:** Department of Diagnostic Medicine/Pathobiology, College of Veterinary Medicine, Kansas State University, ManhattanKS, USA

**Keywords:** short interfering RNA, RNA interference, antiviral, Rift Valley fever virus, nucleoprotein, protein kinase R

## Abstract

Rift Valley fever virus (RVFV) is a mosquito-borne zoonotic pathogen causing severe outbreaks in humans and livestock in sub-Saharan Africa and the Arabian Peninsula. Human infections are characterized by fever, sometimes leading to encephalitis, retinitis, hemorrhagic fever, and occasionally death. There are currently no fully licensed vaccines or effective therapies for human use. Gene silencing mediated by double-stranded short interfering RNA (siRNA) is a sequence-specific, highly conserved mechanism in eukaryotes, which serves as an antiviral defense mechanism. Here, we demonstrate that siRNA duplexes directed against the RVFV nucleoprotein can effectively inhibit RVFV replication in human (MRC5 cells) and African green monkey cells (Vero E6 cells). Using these cells, we demonstrate that individual or complex siRNAs, targeting the RVFV nucleoprotein gene completely abrogate viral protein expression and prevent degradation of the host innate antiviral factor, protein kinase R (PKR). Importantly, pre-treatment of cells with the nucleoprotein-specific siRNAs markedly reduces the virus titer. The antiviral effect of the siRNAs was not attributable to interferon or the interferon response effector molecule, PKR. Thus, the antiviral activity of RVFV nucleoprotein-specific siRNAs may provide novel therapeutic strategy against RVFV infections in animals and humans.

## Introduction

Rift Valley fever virus (RVFV) is a mosquito-borne, zoonotic pathogen that causes Rift Valley fever (RVF) in humans and livestock in sub-Saharan Africa and the Arabian Peninsula (Flick and Bouloy, 2005). The virus is classified as a Category A pathogen and a select agent by the National Institutes of Health (NIH) and the Centers for Disease Control and Prevention (CDC). For decades, RVFV has been associated with epizootics in animals and epidemics in humans in Africa and since 2000, also in the Arabian Peninsula. For example, in 1987, an epidemic of RVF occurred in the Senegal River basin of southern Mauritania and adjacent northern Senegal resulting in 232 human deaths in Mauritania alone, including high rates of abortion in sheep and goats (Jouan et al., 1989). In 2000, for the first time, a RVF outbreak occurred in Saudi Arabia and Yemen resulting in 245 human deaths and the loss of thousands of sheep and goats (Shoemaker et al., 2002). Apparently, the first known human fatality due to RVF was recorded in 1934 in a laboratory worker in the USA soon after the initial isolation of the virus in 1931 (Schwentker and Rivers, 1934). Human RVFV infections are characterized by fever, which may progress to encephalitis, retinitis, hemorrhagic fever, and death (Ikegami and Makino, 2011; LaBeaud et al., 2015). There are no therapies approved for human use. Significant efforts have been directed at vaccine development including development of ΔNSs-ΔNSm recombinant RVFV (Bird et al., 2011) and recombinant RVFV *G*n/*G*c (Faburay et al., 2016) vaccines. Although both vaccines have shown to be safe and efficacious in animals, neither is approved for human use; and in the case ΔNSs-ΔNSm recombinant RVFV, which is a modified live attenuated virus, there are concerns about its use in non-endemic areas. Meanwhile, higher human case fatality rates, ranging from 20% to even 50%, have been reported in recent outbreaks in Africa (Nguku et al., 2010; Heald, 2012; Nanyingi et al., 2015). With the potential of spread of RVFV to non-endemic areas, as well as its potential as a bioterror agent (Mandell and Flick, 2010), the development of effective countermeasures against this pathogen is urgently needed.

RNA interference is recognized as one of the most promising platform for the development of therapeutics against viral pathogens. It represents an endogenous mechanism employed by many organisms used to silence the expression of genes that control various events in a cell (Elbashir et al., 2001) and also provides antiviral activity (Gitlin et al., 2002). Its high efficiency (Bertrand et al., 2002), specificity (Brummelkamp et al., 2002; Xia et al., 2004), and broad applicability could be harnessed to develop a powerful new therapeutic approach for many infections (Haasnoot and Berkhout, 2006; Medarova et al., 2007). The search for novel antiviral agents has led to short interfering RNAs (siRNAs), which act via sequence-selective inhibition of viral replication (Haasnoot et al., 2003; Haasnoot and Berkhout, 2006).

Rift Valley fever virus contains single-stranded, negative, segmented RNA genome that encodes four structural proteins, the nucleoprotein (N), the glycoproteins *G*n and *G*c, and the L polymerase; two non-structural proteins, NSs and NSm, and a 78-kDa protein of unknown function (Gerrard and Nichol, 2007; Won et al., 2007). Although both NSs and NSm are dispensable for RVFV replication *in vitro* and *in vivo* (Ikegami et al., 2006; Gerrard et al., 2007; Bird et al., 2008), both proteins play a critical role in viral pathogenesis. NSm acts as an anti-apoptotic protein (Won et al., 2007), whereas NSs, the major viral virulence factor, inhibits host innate immune response (Bouloy et al., 2001) through generalized downregulation of RNA transcription, including suppression of interferon-β (IFN-β) (Billecocq et al., 2004; Le May et al., 2004, 2008) and degradation of protein kinase R (PKR) (Habjan et al., 2009; Ikegami et al., 2009a). PKR is a host-encoded pattern recognition receptor with innate antiviral activity (Ikegami et al., 2009b). The RVFV N protein is associated with the viral genomic RNA and together with the L protein makes up the ribonucleoprotein (RNP) complex, which is responsible for RNA transcription and replication of the RVFV genome. Both genes are highly conserved (Ikegami, 2012). Therefore, the N and L proteins represent ideal targets for developing antiviral interventions using siRNAs. We therefore hypothesized that posttranscriptional silencing of the RVFV N and L genes will have significant effects RVFV replication. Using the attenuated RVFV strain MP12 (Caplen et al., 1985), classified as a BSL-2 pathogen, we assessed the effect of posttranscriptional silencing of the N and L polymerase genes on RVFV replication.

## Materials and Methods

### Cells, Virus, and Virus Titrations

Human lung fibroblast cells (MRC-5; ATCC, Manassas, VA, USA) and African Green Monkey kidney cells (Vero E6 cells) were propagated in modified Eagle medium (MEM) supplemented with 10% fetal bovine serum (FBS) at 37°C with 5% CO_2_. MP12 is an attenuated RVFV strain (Caplen et al., 1985) and was used as the RVFV strain for all cell infections. The MP12 virus carries a functional virulence gene, NSs, and sequence alignment of its nucleoprotein gene shows 100% identity to the parent wild-type RVFV strain, ZH548 (unpublished data). MP12 virus titers were quantified in Vero E6 cells using standard protocols (Faburay et al., 2014) and titers were expressed as plaque forming units per ml (pfu/ml).

### Recombinant Plasmid Constructs

The plasmid pAcGFP (Takara Clontech, Mountain View, CA, USA) was linearized with the restriction enzyme *Hind*III (New England Biolabs, Ipswich, MA, USA). RVFV N coding sequence, based on the RVFV strain ZH548, was amplified from the donor plasmid, pFastBacNP (Faburay et al., 2013) using gene-specific primers. The primers were designed using the primer design software (Takara Clontech) that allowed directional cloning of the PCR amplicon into a linearized pAcGFP using the In-Fusion Cloning Plus kit (Takara Clontech). The primers used were JAR121F 5′-TCTCGAGCTCAAGCTTATGGACAACTATCAAGACCTTGCGATCC-3′ and JAR122R 5′-GCAGAATTCGAAGCTTGGCTGCTGTCTTGTAAGCCTGAGCG-3′ (vector sequences are underlined). PCR was performed using proof-reading DNA polymerase, *Pfx*50 DNA polymerase (Life Technologies, Carlsbad, CA, USA) using reaction conditions as specified by the manufacturer. The PCR amplicons were purified using a PCR purification kit (Qiagen, Valencia, CA, USA) and cloned into pAcGFP according to the manufacturer’s instructions to create the recombinant expression vector, pAcGFP-N. The RVFV N sequence was cloned without the stop codon and in-frame with the coding sequence of the Green Fluorescent protein (GFP) to allow expression of a chimeric RVFV N-GFP fusion protein. The cloning reaction was transformed into chemically competent *E. coli* Max Efficiency (Life Technologies, Carlsbad, CA, USA) and transformants were selected on Luria-Bertani (LB) agar plates containing kanamycin (30 μg/ml). Recombinant plasmids were purified from overnight *E. coli* cultures using a Miniprep kit (Qiagen, Valencia, CA, USA). The presence and integrity of RVFV N-GFP chimeric sequences were determined by restriction enzyme analysis and DNA sequencing.

### Design and *In vitro* Screening of siRNAs in Cotransfection Experiments

Several siRNA duplexes targeting different loci within the RVFV N (si46N, si252N, si605N, si476N) and L polymerase (si5849L) mRNA transcripts were designed using a conventional siRNA design algorithm (**Table 1**). Additionally, siRNAs targeted at GFP (si475GFP) and *Renilla luciferase* (si689RL) genes were constructed for use as positive knockdown and scrambled negative controls, respectively (**Table 1**). The Basic Local Alignment Search Tool (National Center for Biotechnology Information, Bethesda, MD, USA) was used to exclude siRNAs with sequence homology to any human reference mRNAs of 16 or more contiguous bases within the core duplex. The siRNA duplexes were synthesized by Integrated DNA Technologies (Coral Ville, IA, USA). Screening of siRNAs to assess knockdown of gene expression was performed using a Lipofectamine 2000 (Life Technologies, Carlsbad, CA, USA) cotransfection protocol. Briefly, individual siRNA duplexes were reconstituted in DNase and RNase-free water to a stock concentration of 20 or 40 μM. For transfection, MRC5 cells were seeded and grown overnight in 12-well plates to 80–90% confluency. Thereafter, 150 ng of each plasmid, pAcGFP-N or intact pAcGFP control plasmid, was mixed with 10 pmol of the respective RVFV N-specific siRNAs, si46N/si605N mixed, or individually, si475GFP (GFP positive control) or si689RL (*Renilla luciferase* negative scrambled control) in Opti-MEM reduced serum medium (Life Technologies, Carlsbad, CA, USA). After 20 min incubation, the reaction mixture was added dropwise to MRC5 cells in Opti-MEM reduced serum medium. After 6 h post transfection, the reduced serum medium was replaced with complete cell culture medium and cells incubated in a humidified incubator at 37°C with 5% CO_2_ for 48 h. Knockdown of target gene expression was assessed by fluorescent microscopy (Nikon, Eclipse TE2000-S) and Western blot analysis.

**Table 1:** Sequences of small interfering RNAs used for knockdown of target gene expression.

siRNA ID	Sense	Antisense	Target gene
si46N	ggaccgcaaugagauugaauu	uucaaucucauugcgguccuu	RVFV nucleoprotein
si605N	gcagugaauagcaacuuuauu	uaaaguugcuauucacugcuu	RVFV nucleoprotein
si252N	cucucaucaacaaguauaauu	uuauacuuguugaugagaguu	RVFV nucleoprotein
si476N	gacuaucuaagggcaauauuu	auauugcccuuagauagucuu	RVFV nucleoprotein
si5849L	gucggaucuauguucucauuu	augagaacauagauccgacuu	RVFV RNA polymerase
si475GFP	gaauggcaucaaggugaacuu	guucaccuugaugccauucuu	Green fluorescent protein
si689RL	ccugacguuguacaaauuguu	caauuuguacaacgucagguu	*Renilla luciferase*


### Western Blot

Western blot analysis was performed to assess siRNA-mediated knockdown of target protein expression (N and GFP). Also, inhibition of cellular PKR (a constitutively expressed host antiviral innate immune response factor) expression was assessed as a function of intracellular RVFV replication. Beta actin expression was used as a loading control. Briefly, MRC5 and Vero cells were washed with PBS pH 7.4 and the cells resuspended in lysis buffer (PBS pH 7.4 containing 1% Triton X-100 and 1x Roche Complete Protease Inhibitor). Approximately, 5 μg of total protein lysate was resolved in 12% Bis-Tris polyacrylamide gel (Life Technologies, Carlsbad, CA, USA). The proteins were transferred by electroblotting onto PVDF membranes according to standard protocols. After blocking for 1 hr in PBS pH 7.4 containing 0.1% Tween20 and 3% bovine serum albumin, the blots were probed with either mouse anti-RVFV N (R3-ID8; 1: 2,000) (BEI Resources, NIH, Manassas, VA, USA), anti-GFP (1:300) (Santa Cruz Biotechnology, Dallas, TX, USA), anti-PKR (1:200) or anti-beta actin (1:200; Santa Cruz Biotechnology, Dallas, TX, USA) monoclonal antibodies for 1 hr. The membranes were washed and then incubated for 1 hr with goat anti-mouse-HRP conjugated secondary antibody (1:5,000) (Santa Cruz Biotechnology, Dallas, TX, USA) and specific reactivity was detected using enhanced chemiluminescent (ECL) detection system (GE Healthcare, Buckinghamshire, UK).

### Determination of siRNA Effects on RVFV Protein Expression

Vero E6 cells were seeded overnight in 12-well plates at 1 × 10^6^ cells per well so that they are 80–90% confluent the next day at the time of transfection. On the day of transfection, the old medium was replaced with fresh medium. Transfection mixtures were prepared using RNAiMax reagent (Life Technologies, Carlsbad, CA, USA) in Opti-MEM reduced serum medium using 50 nM (final concentration) of each siRNA. This was empirically determined to be an optimal siRNA concentration that inhibits viral replication without noticeable effect on cell viability (data not shown). To assess inhibition of RVFV nucleoprotein expression in Vero cells, the following siRNAs were tested in duplicate wells: (i) si605N/si46N/si252N/si476N, (ii) si605N, (iii) si46N, (iv) si605N/si5849L, (v) si5849L, (vi) si475GFP and (vii) si689RL (scrambled negative control). At 24 h post transfection, cells were infected with MP12 virus at multiplicity of infection (MOI) of 2. After 1 h adsorption period, the viral inoculum was removed and replaced with 1 ml complete culture medium (MEM supplemented with 10% FBS), and the cells incubated at 37°C for 48 h. Cell lysates were prepared as described above and analyzed by Western blot.

### Determination of siRNA Effects on Prevention of Protein Kinase R Degradation

The RVFV MP12 carries a functional NSs gene, a major RVFV virulence factor, responsible for inhibition of host innate immune response including degradation of PKR (Billecocq et al., 2008; Habjan et al., 2009; Ikegami et al., 2009a). Thus viral degradation of cellular PKR was assessed as a parameter to detect RVFV replication in human cells and its response to siRNA treatment. MRC5 cells were seeded overnight in 12-well plates at 1 × 10^6^ cells per well so that they are 80–90% confluent the next day at the time of transfection. On the day of transfection, the old medium was replaced with fresh medium. Transfection mixtures were prepared with RNAiMax reagent (Life Technologies, Carlsbad, CA, USA) in OptiMEM reduced serum medium to contain 50 nM (final concentration) of each siRNA as described above. At 24 h post transfection, cells were infected with MP12 virus at a MOI of 1. After 1 h adsorption period, the viral inoculum was removed and replaced with 1 ml complete culture medium, and the cells were incubated at 37°C for 48 h. Cell lysates were prepared as described above and analyzed for expression of PKR by Western blot.

### Determination siRNA Effects on RVFV Replication

#### Quantitative RT-PCR

To assess siRNA inhibition of RVFV RNA replication, viral RNA was extracted from the culture supernatants using Qiagen Viral RNA extraction kit (Qiagen, Valencia, CA, USA). Briefly, Vero E6 cells were seeded overnight at 1 × 10^6^ cells per well so that they are 80–90% confluent the next day at the time of transfection. Wells were transfected in duplicate with si605N, si46N, si252N, si476N, and siPooledN (si605N/si46N/si252N/si46N) as described above. At 48 h post transfection, cells were infected with MP12 virus at MOI of 0.1. After 1 h adsorption, the virus inoculum was removed and replaced with complete cell culture medium. At 24 h post infection, supernatants were collected and aliquots were used to determine viral RNA replication by qRT-PCR and virus replication by plaque assay (described below). Briefly, a quantitative RT-PCR (qRT-PCR) targeting the L segment was performed (Faburay, unpublished). A SuperScript III One-step RT-PCR protocol with forward primer BJF 5′-CTT AGC TGA CAA GAC TGA CAG AC-3′ and reverse primer BJR 5′-GTA CCT ATA AAC CAT CTC CTC TGC-3′ and Taqman Probe BJP FAM 5′-AGG GGA GAT GAA AGA GGT GCA TTC CAG GCT-3′-IABKFQ (Iowa Black) was used. The assay was performed using the following reaction conditions: 50°C for 15 min, and then 35 times 95°C for 2 min and 60°C for 15 s, using the CFX96 Real-Time System (Bio-Rad, Hercules, CA, USA).

#### Flow Cytometry

To further assess the effect of siRNAs on viral replication, flow cytometry analysis was performed. Briefly, cells were seeded and transfected with the various siRNAs (si605N, si46N, si252N, si476N, and siPooledN (si605N/si46N/si252N/si46N) as described above. siRNA untreated but virus-infected (virus control) cells, and untreated, non-infected cells were included as relevant controls. At 48 h post transfection, cells were infected with MP12 virus at a MOI of 0.1. After 1 h adsorption, the virus inoculum was removed and replaced with complete cell culture medium. At 24 h post infection, cells were harvested using Accutase (MP Biomedicals, Solon, OH, USA) and were used for flow cytometry analysis. Briefly, cells were pelleted by centrifugation at 5,000 × g for 10 min and then fixed in 4% paraformaldehyde for 30 min. The cell pellets were resuspended in PBS pH 7.4 containing 10 mM glycine and incubated overnight at 4°C. Thereafter, the cells were stained by incubating with mouse anti-RVFV N monoclonal antibody (R3-4D8) (1:100 dilution; BEI Resources, NIH, Manassas, VA, USA) in permeabilized buffer (PBS, 7.4 containing 0.1% saponin, 20 mm EDTA, 0.02% sodium azide, 2% FBS) for 1 h at 4°C. Following two washing steps, cells were incubated with Alexa Fluor-488 conjugated goat-antimouse IgG antibody (5 μg/ml final concentration) (Molecular Probes, Thermo Fisher Scientific, Eugene, OR, USA) for 30 min at 4°C. Cells were analyzed by flow cytometry on BD LSR Fortessa X-20 using a 525/50 Band Pass Filter for the FITC detector off a 488 nm blue laser.

#### Virus Titration by Plaque Assay

Virus titrations were performed in Vero E6 cells by plaque assay according to previously described protocols (Faburay et al., 2014) using supernatants collected from the experiments described above for qRT-PCR. Briefly, 10-fold serial dilutions of the supernatants from the various siRNA treatments were made and used to infect monolayers of overnight cultures of Vero E6 cells in 6-well plates. After 1 h adsorption of the virus at 37°C and 5% CO_2_, the viral inoculum was removed and the monolayer overlaid with 0.9% agarose. The plates were incubated at 37°C and 5% CO_2_ for 4 days. After fixing with 10% formalin for 3 h (Faburay et al., 2014), monolayers were stained with 0.5% crystal violet and plaques counted and quantified according to standard protocol. Titers were expressed as plaque forming units per ml (pfu/ml).

### Statistical Analysis

Differences in response values between siRNA treated and untreated controls were analyzed for statistical significance using Graphpad Prism 6. An unpaired *t*-test was used to compare mean response values between various siRNA treatments, as well as compare the mean values of the siRNA treatments and the controls. A *P*-value of ≤0.05 was considered statistically significant.

## Results

### Knockdown of GFP and RVFV N Protein Expression Using siRNAs in pAcGFP Transfected MRC-5 Cells

**FIGURE 1:**
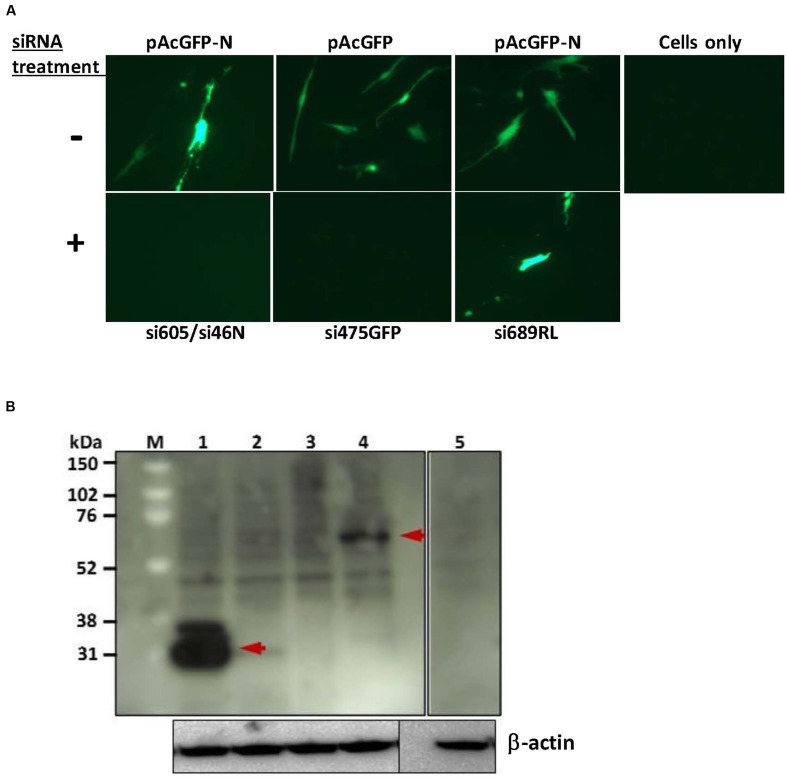
**siRNA-knockdown of exogenous gene expression in MRC5 cells.** **(A)** Fluorescence analysis shows inhibition of pAcGFP-N and pAcGFP plasmid expression. RVF N-specific si605N/si46N cotransfection (+) inhibited expression of GFP-N fusion protein in contrast to the untreated control (-); GFP-specific si475GFP cotransfection (+) inhibited expression of the green fluorescent protein. si689RL, the scrambled non-specific *Renilla luciferase*-siRNA, shows no inhibition of GFP-N protein expression. **(B)** Western blot analysis of siRNA knockdown of exogenous protein expression. *Lane 1*: expression of GFP (without siRNA treatment; arrow shows an estimated 27 kDa molecular weight protein); *lane 2*: knockdown of GFP expression with si475GFP; *lane 3*: knockdown of GFP-N fusion protein with si605N/si46N; *lane 4*: expression of GFP-N fusion protein (without siRNA treatment; arrow shows an estimated 54 kDa molecular weight protein); lane 5: cells only control. β-actin serves as loading control.

Knockdown of the expression of the RVFV N-GFP fusion protein encoded by the plasmid pAcGFP-N was performed in MRC-5 cells using the RVFV nucleoprotein-specific siRNAs si605N/si46N. Co-transfection of the pAcGFP-N plasmid with the siRNAs si605N/si46N resulted in inhibition of N-GFP fusion protein expression as demonstrated by the absence of specific GFP fluorescence (**Figure 1A**) and the fusion protein-specific band in Western blot analysis (**Figure 1B**). Treatment with the positive control siRNA, si475GFP, targeting the GFP gene, abrogated expression or fluorescence of the GFP-N fusion fusion protein (**Figures 1A,B**). In contrast, GFP fluorescence was observed in all mock-treated cells transfected with the recombinant plasmids pAcGFP-N or pAcGFP (**Figure 1A**). Treatment of the MRC5 cells with the scrambled negative control siRNA, si689RL, did not have any effect on fusion protein expression as demonstrated by clear expression of the N-GFP fusion protein (**Figure 1A**).

### Inhibition of RVFV Nucleoprotein Expression in MP12-Infected Vero Cells

**FIGURE 2:**
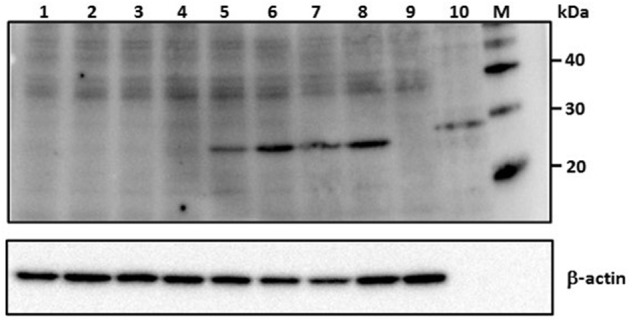
**RVFV siRNA-specific inhibition of viral nucleoprotein expression in Vero E6 cells.** Numbers above the panel depict various siRNA treatments. *Lane 1*: si605N/si46N/si252N/si476N (siPooledN; RVFV N-specific siRNAs); *lane 2*: si605N; *lane 3*: si46N; *lane 4*: si605N/si5849L; *lane 5*: si5849L (RVFV L-segment specific siRNA); *lane 6*: si475GFP (GFP-specific siRNA); *lane 7*: si689RL (*Renilla luciferase*-siRNA); *lane 8*: untreated virus control; *lane 9*: uninfected cell only control; *lane 10*: recombinant RVFV N protein control (∼30 kDa). *Lanes* 5, 6, 7, 8: show expression of viral nucleoprotein (∼27 kDa). β-actin serves as loading control. M, molecular weight marker.

Viral nucleoprotein expression in Vero E6 cells infected with MP12 RVFV was completely abrogated by all the RVFV N-specific siRNA treatments (lanes 1–4; **Figure 2**). Specifically, pretreatment with a complex pool of siRNAs, si605N/46N/252N/476N (siPooledN), si605N/si5849L or with individual siRNA duplexes, si605N or si46N, completely abrogated RVFV nucleoprotein expression following RVFV infection. However, when cells were treated with siRNA si5849L alone, which targets the viral L polymerase gene, no inhibition of viral nucleoprotein expression was observed (**Figure 2**). Similarly, pretreatment with non-specific scrambled siRNAs, si475GFP, and si689RL, specific to transcripts of GFP and *Renilla luciferase*, respectively, did not inhibit nucleoprotein expression (**Figure 2**). Untreated MP12 infected cells showed distinct nucleoprotein expression (**Figure 2**, lane 8). Uninfected and siRNA-untreated cells controls did not show any nucleoprotein expression (**Figure 2**, lane 9).

### siRNA Treatment Prevents Degradation of Protein Kinase R

**FIGURE 3:**
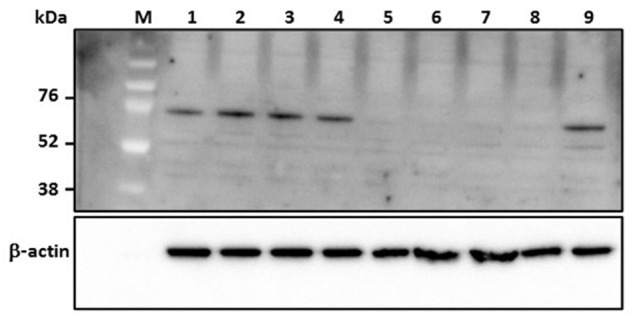
**Analysis of degradation of protein kinase R (PKR) in human cells (MRC5) in response to RVFV replication.** Numbers above the panel depict various siRNA treatments. *Lane* 1: si605N/si46N/si252N/si476N (siPooledN; RVFV N-specific siRNAs); *lane* 2: si605N; *lane* 3: si46N; *lane* 4: si605N/si5849L; *lane* 5: si5849L (RVFV L-segment specific siRNA); *lane* 6: si475GFP (GFP-specific siRNA); *lane* 7: si689RL (*Renilla luciferase*-specific siRNA); *lane* 8: virus control (untreated); *lane* 9: uninfected cell only control. *Lanes* 1, 2, 3, 4: show inhibition of PKR degradation due to RVFV-specific siRNA treatment as indicated by detection of a 68 kDa specific cellular protein. β-actin serves as loading control. M, molecular weight marker.

Protein kinase R is a host-encoded interferon response effector molecule that is subjected to degradation by the RVFV non-structural protein NSs upon viral infection; this allows NSs to suppress the host innate immune responses (Habjan et al., 2009). The NSs coding sequence of RVFV strain MP12 is functionally active as demonstrated by degradation of constitutively expressed PKR in MP12-infected MRC5 cells (data not shown). Thus, expression of PKR in MP12-infected MRC-5 cells treated with RVFV N-specific siRNA should result in inhibition of RVFV replication. Treatment with RVFV-specific siRNAs, si605N/si46N/si252N/si476N (siPooledN), si605N/si5849L, si605N or si46N, significantly inhibited degradation of PKR in MP12- infected cells as demonstrated by detection of the PKR-specific 68 kDa protein in Western blot analysis (**Figure 3**). When si5849L, targeting the L polymerase gene, was used, degradation and loss of PKR in MP12-infected cells was observed. Similarly, treatment with non-specific siRNAs, si475GFP, and si689RL, did not protect PKR from NSs-mediated degradation (**Figure 3**).

### siRNA Treatment Inhibits RVFV MP12 Replication

**FIGURE 4:**
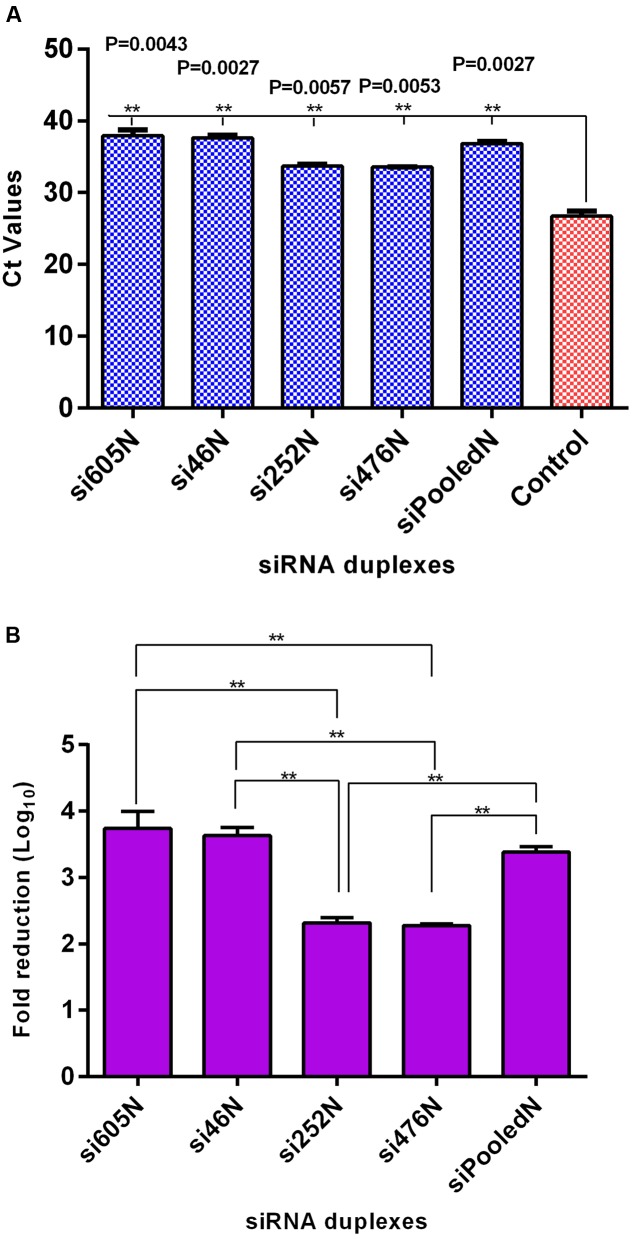
**qRT-PCR analysis of RVFV replication in response to RVFV-specific siRNA treatment.** **(A)** *C*t values of viral RNA amplification using supernatants from the various treatments; the Ct values of the siRNA treatments differed significantly from the untreated virus control (*P* < 0.05). **(B)** Illustrates log_10_ fold reduction in viral titers relative to the untreated virus control. si605N and si46N treatments show the highest fold-reductions. Asterisks (^∗∗^) above horizontal bars compare differences between treatments and denote significant differences. Fold reductions in viral titers of si605N or si46N were not different from siPooledN viral titers (*P* > 0.05).

**FIGURE 5:**
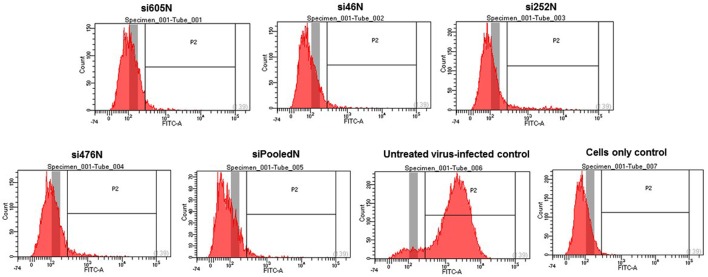
**Flow cytometry analysis of RVFV replication in response to RVFV-specific siRNA treatment.** Only background fluorescence is detectable in the various siRNA treatments in contrast to the intense fluorescence detected in positive gate of the virus-infected control. A cell only control (untreated and uninfected) is also shown.

**FIGURE 6:**
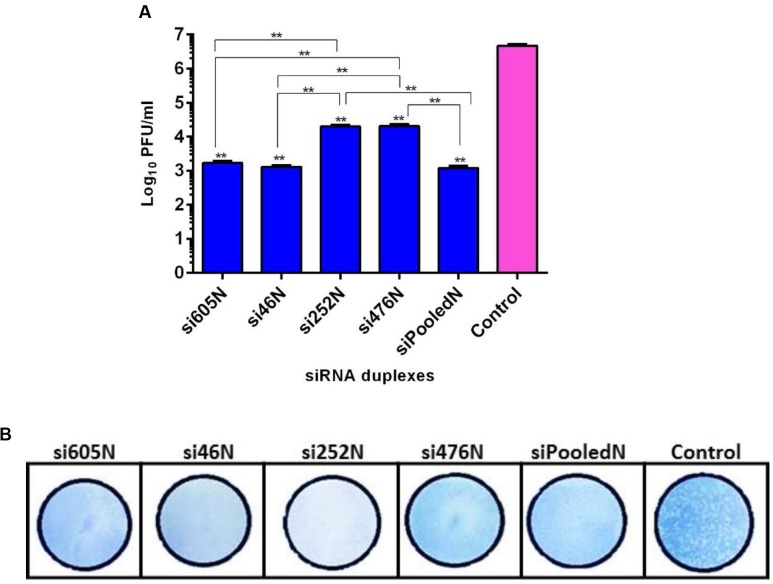
**Quantitation of RVFV replication by plaque assay.** **(A)** shows titers of viral progeny detected in the supernatants of the various siRNA treatments. Asterisks (^∗∗^) directly above the bars indicate significant differences compared to the virus control (*P* < 0.05). Asterisks (^∗∗^) above horizontal lines compare differences between treatments and denote significant differences. **(B)** Demonstrates inhibition of viral replication using 10^4^-fold dilution of culture supernatants from the various treatments. In contrast to the untreated virus control, no plaque could be isolated from the supernatants of the siRNA treatments.

As shown above, RVFV N-specific siRNAs inhibit viral nucleoprotein expression and prevent degradation host encoded PKR. Here, their potential to inhibit viral replication was assessed using qRT-PCR, flow cytometry and virus plaque assays. qRT-PCR cycle threshold (*C*t) values obtained in cells infected with MP12 and treated with RVFV N-specific siRNAs were significantly higher when compared to the untreated control cells (*P* < 0.05) (**Figure 4A**). The amount of viral RNA produced after treatment with N-specific siRNAs, individually or as pooled complex, were at least 3 logs lower when compared to the untreated MP12-infected control (*P* < 0.05). Specifically, si605 and si46 displayed the highest fold reductions in virus replication of log_10_ 3.74 and 3.63, respectively (**Figure 4B**). Flow cytometry analysis confirmed these results. Only background fluorescence was detectable in all N-specific siRNA treatments indicating effective inhibition of MP12 replication. In contrast, a strong positive fluorescence was detected in the untreated virus-infected control cells (**Figure 5**). Quantitation by plaque assay confirmed the above results. Production of viral progeny was significantly inhibited by the N-specific siRNA treatments when compared to the untreated infected control (*P* < 0.05) (**Figure 6A**). Meanwhile, si605N, si46N, and siPooledN exhibited stronger inhibition than si252N and si476N (**Figure 6A**). The magnitude of inhibition of virus replication was demonstrated by the absence of viral plaques in a 10^4^ dilution of culture supernatants treated with N-specific siRNAs (**Figure 6B**). In contrast, viral plaques were easily detected in the untreated infected control (**Figure 6B**).

## Discussion

Since the first reported outbreak of RVF in 1931 (Daubney and Garnham, 1931), RVFV has been associated with periodic outbreaks of hemorrhagic fever in humans in Africa and the Arabian Peninsula (Balkhy and Memish, 2003; Anyangu et al., 2010; Sow et al., 2014; Boushab et al., 2015) with increasing case fatality rates (Heald, 2012). There is currently no effective therapy for RVFV infection in humans and animals; attempts have been made to treat the disease with unproven antiviral compounds such as ribavirin (Huggins, 1989). However, ribavirin has been shown to cause serious undesirable side effects following treatment (Snell, 2001; Scharton et al., 2014). Thus, current therapies are palliative in order to relieve patients of the symptoms of the disease. Considering the lack of good therapeutic approaches for RVF, we aimed at developing effective therapeutic intervention strategies for RVF. Therefore, this study was carried out to investigate the potential application of RNAi as a therapeutic approach to treat RVF in humans and animals.

We assessed the impact of posttranscriptional siRNA silencing of the RVFV nucleoprotein and the RNA-dependent RNA polymerase L on viral replication using an *in vitro* cell culture model and the MP12 strain of RVFV. All 4 siRNA constructs targeting the viral nucleoprotein gene/mRNA exhibited significant inhibition of RVFV protein expression and replication. In contrast, si5849L, targeting the viral L polymerase exhibited no detectable effect on RVFV protein expression (**Figure 2**) and viral replication (data not shown). The reason for the non-inhibition is unknown, and consequently, siRNA si5849L was not studied further. The RVFV N gene represents a good target for posttranscriptional RNAi silencing (Scott et al., 2012) since the gene is highly conserved among all known strains of RVFV (Sall et al., 1997), including the attenuated strain MP12 used in this study. Although MP12 is reported to contain mutations in all 3 of its genome segments (Saluzzo and Smith, 1990; Ikegami et al., 2015), sequence analysis revealed the absence of nucleotide mutations in the N gene, indicating a 100% identity with the parent wild-type virus ZH548 (data not shown). Similarly, the MP12 N nucleotide sequence has a high homology with N genes from other wild-type RVFV isolates such as ZH501 (Rossi and Turell, 1988), SA01 (Miller et al., 2002), and Kenya 128B-15 (Sang et al., 2010) of 99.59, 97.15, and 97.15%, respectively (data not shown). This strongly suggests that the siRNA constructs, especially the pooled cocktail formulation, will have inhibitory effect on wild-type RVFV strains.

Protein kinase R is a host-encoded interferon response effector molecule constitutively expressed in mammalian cells. Upon RVFV infection, it is degraded by the viral non-structural protein NSs acting to inhibit host anti-viral innate immune response (Habjan et al., 2009; Ikegami et al., 2009a,b). Although the MP12 virus encodes attenuated M- and L-segments, it carries a virulent NSs gene (Billecocq et al., 2008), thereby retaining the inherent capacity to degrade constitutively expressed PKR in infected mammalian cells. Thus, we indirectly examined the inhibitory effect of the N-specific siRNAs on RVFV replication by assessing the expression of PKR as a function of RVFV replication. Treatment of infected cells with the RVFV N-specific siRNAs completely inhibited degradation of PKR, indicating the effectiveness of the siRNA constructs at inhibiting viral replication and consequently NSs activity (**Figure 3**). In addition, the antiviral effect of the N-specific siRNAs is not attributable to interferon or interferon response effector molecules, since some studies were carried out in an interferon deficient cell line, Vero E6 cells.

Using multiple quantitative assays, e.g., qRT-PCR, flow cytometry and virus plaque assay, we further demonstrated significant inhibition of MP12 replication following treatment with the RVFV N-specific siRNAs (**Figures 4–6**). All N-specific siRNAs, including the pooled formulation (siPooledN), significantly reduced virus replication as determined by qRT-PCR (*P* < 0.05) (**Figure 4A**). The levels of viral RNA were reduced by at least 3 logs in comparison to the untreated infected control (**Figure 4B**), with si605N, si46N and siPooledN exhibiting significantly higher reductions compared to si252N and si476N (P < 0.05) (**Figure 4B**). A similar picture was seen using flow cytometry analysis, whereby only the untreated virus-infected control displayed RVFV-specific, positive fluorescence (**Figure 5**). All N-specific siRNA treatments did not result in RVFV protein expression, indicating effective suppression of viral replication. The above findings were confirmed by virus plaque assays (**Figure 6A**) in which the levels of inhibition of virus replication was quantified. The complete absence of viral plaques in a 10^4^ dilution of culture supernatants treated with the N-specific siRNAs (**Figure 6B**) confirms the efficacy of this treatment regimen.

The development of resistance by RNA viruses to antiviral drugs (Hayden and de Jong, 2011) is a global concern in the fight to prevent potential viral epidemics or pandemics. RNAi is considered a promising platform for therapeutic drug development to overcome these challenges. Unlike conventional therapeutic drugs, antiviral resistance to siRNAs due to single nucleotide mutations could be easily addressed by redesigning new constructs or using pooled formulations that target multiple loci within the mRNA transcript. Once sequence data of a new emergent strain are available, it is estimated that clinical grade siRNA products can be produced in as little as 8 weeks if deemed necessary (Thi et al., 2015). Thus, the application of RNAi as a therapeutic option for hemorrhagic viral diseases should be given serious consideration.

The recent outbreak of Ebolavirus in West Africa claimed the highest number of human lives in the history of Ebolavirus disease (ECDC, 2014). To combat the disease, several post-exposure interventions including lipid nanoparticle siRNA treatment, which has shown 100% protection in rhesus monkeys against lethal Ebola virus challenge, have been used (Geisbert et al., 2010; Thi et al., 2015). Although the Ebola siRNA treatment, at the time, was not fully vetted, the dire circumstances of the patients inflicted with the lethal disease provided enough justification for the US Food and Drugs Administration (FDA) to allow “compassionate use” of the drug to treat Ebola patients (Grens, 2014; Bishop, 2015). In 2015, the Ebola siRNA treatment underwent a Phase II clinical study in Ebola virus disease patients in Sierra Leone, West Africa (Dunning et al., 2016). In view of the societal, economic, and other disruptions, which can be associated with outbreaks of hemorrhagic viral infections in humans (Faburay, 2015; Kupferschmidt, 2015; Nanyingi et al., 2015), consideration should be given to RNAi as a therapeutic platform in outbreak situations. In the last decades, outbreaks of RVF in various countries in Africa, e.g., Kenya (Nguku et al., 2010), South Africa (Archer et al., 2013), Mauritania (Sow et al., 2014; Boushab et al., 2015), and Saudi Arabia (Al-Hazmi et al., 2003; Himeidan et al., 2014), caused significant human deaths and economic hardships for farmers.

## Conclusion

We have demonstrated the feasibility of RVFV nucleoprotein-specific siRNAs to inhibit RVFV replication, suggesting the utility of this technology as a potential therapeutic tool for RVFV infection. In future studies, we plan to assess the antiviral effect of these siRNAs with wild-type RVFV isolates and in various experimental animal models including murine, ruminant, and non-human primates. Furthermore, we might examine co-administration of siRNAs in conjunction with subunit vaccines; this approach could provide protection during the period of the immunity gap before the vaccine provides efficient protection (Kim et al., 2015). Adopting such a vaccination scheme could enhance vaccine efficacy and improve the overall efficiency of RVF vaccination programs.

## Author Contributions

BF and JR contributed to the conception of the study. BF designed the siRNAs, developed and optimized the experimental protocols and performed the experiments. BF and JR analyzed the data and wrote the manuscript.

## Conflict of Interest Statement

The authors declare that the research was conducted in the absence of any commercial or financial relationships that could be construed as a potential conflict of interest.
